# Children, Sexual Abuse and the Emotions of the Community Health Practitioner in England and Wales (1970-2000)

**DOI:** 10.1093/jhmas/jrad024

**Published:** 2023-09-23

**Authors:** Ruth Beecher

**Affiliations:** Birkbeck, University of London

**Keywords:** child sexual abuse, emotions, community health practitioners, early recognition, doctors, nurses

## Abstract

A survivor of child sexual abuse felt that doctors missed opportunities to notice her distress when, at fourteen, she had an unexplained illness that lasted for a year. The cause, she wrote, was “explained by Doctors as psychological, but nobody questioned further. WHY??? … If adults don’t listen[,] then we have no one to turn to.” For decades, community health practitioners have been identified as an important group in protecting children from maltreatment, but survivor testimony and agency statistics demonstrate that they rarely receive verbal disclosures or recognize the physical or behavioural warning signs of sexual abuse. The accounts we have of the 1980s tell of swiftly heightening professional awareness, followed by a visceral backlash in the latter part of the decade that discouraged practitioners from acting on their concerns. This article uses trade and professional journals, training materials, textbooks and new oral histories to consider why community-based doctors and nurses have struggled to notice and respond to the sexually abused child. It will argue that the conceptual model of child sexual abuse community health practitioners encountered in the workplace encouraged a mechanical and procedural response to suspicions of abuse. In a highly gendered and contested workplace, practitioners' feelings about how survivors, non-abusing family members and perpetrators should be understood were rarely debated in training or in practice. The emotional cost to the practitioner of engagement with sexual abuse, and their need for spaces of reflexivity and structures of support, were ignored.

## Introduction

In 1995, hundreds of adult survivors of child sexual abuse wrote to the National Commission of Inquiry into the Prevention of Child Abuse. Established in 1994 and chaired by a senior barrister, Lord Williams of Mostyn, it followed on the heels of numerous inquiries into child deaths over the previous two decades. Public inquiries have been extensively used in the United Kingdom throughout the twentieth century to investigate issues of public concern, but this was slightly unusual. It was set up at the initiative of the National Society for the Prevention of Cruelty to Children (NSPCC) and it took a broader stance than previous similar inquiries, emphasizing wider strategies for the prevention of further abuse. It was distinctive too in the way it reached out beyond “expert” testimony to the victims and survivors of all forms of child abuse. Appeals in magazines, tabloid newspapers, and breakfast television programmes told audiences: “Here’s your chance to help other abused children.”^1^

Survivors responded to the call to “bear witness” in large numbers; the inquiry received more than a thousand letters, most from adults who were sexually abused as children. One young adult told the inquiry that as a girl, she had been regularly raped by her male cousin and had been “forever at the doctors with urine infections and other problems ‘down there.’” She didn’t blame the doctor for failing to notice the signs. Instead, she held herself responsible: she was “so terrified” of her cousin’s threats to kill her if she told anyone that "I had probably subconsciously gone out of my way to behave as normally as possible.”^2^ Other survivors were less forgiving about failed opportunities to stop their abuse. A survivor who had a year’s unexplained illness when she was fourteen years old complained that the “cause was explained by Doctors as psychological, but nobody questioned further. WHY??? … Every signal that an abused child gives out has taken tremendous courage and perhaps a lifetime of thought. If adults don't listen[,] then we have no one to turn to.”^3^

For decades, community health practitioners have been identified as an important group in protecting children from abuse, but the sexually abused child has not been generally well served by their family doctor or health visitor. ^4^ Child and adult survivors have testified to this. In letters to the same inquiry, survivors wrote “how they must have been giving off signs, but no one bothered to ask what the matter was.”^5^ Although agency statistics should be assessed cautiously, they tell a similar story.^6^ Analysis of referral sources to child protection services demonstrates that not only have community health practitioners not received verbal disclosures, they have also very rarely recognized the physical or behavioural warning signs of sexual abuse.^7^

Historians have exhumed earlier waves of awareness and amnesia in relation to the sexual violation of children. They have interrogated the role of race and class in what could be named or ignored.^8^ They have asked what symbolic part sexually abused children played as sites for wider ideological debates.^9^ The ways in which children making allegations were treated, the credibility afforded to the physical signs of sexual abuse, and the responses of professionals as well as the state have been examined.^10^ Scholars have paid less attention to those practitioners who have been required by government, their own professionals bodies, and their employers to identify child sexual abuse at the earliest possible point in time. A key group were those working in community health settings, who, since the 1980s, have been expected to be alert to the possibility of child *sexual* abuse specifically. This responsibility included listening carefully and observing closely: was a child attempting to disclose that they had been sexually victimized, were there physical signs or indications through their behaviour that abuse might have occurred? The accounts we have of the 1980s tell of swiftly heightening professional awareness, followed by a visceral “backlash” in the latter part of the decade that portrayed “hysterical” health and social work professionals as zealots who “seized” children from their “innocent” parents.^11^ Although the criticisms were mainly of paediatricians and social workers, the implication was that all sorts of practitioners retreated fearfully from their responsibilities and shut their eyes to potential indicators of abuse.

By focusing on the history of how UK-based community health practitioners encountered ideas about child sexual abuse, this article reveals that even before the apparent “backlash” at the close of the 1980s, general practitioners (GPs) and health visitors were unlikely to make progress in encouraging children to disclose sexual abuse or in spotting the behavioural or physical signs. One reason for this was that practitioners’ feelings about the causes and meanings of child sexual abuse and how victims, non-abusing parents, and perpetrators should be understood were rarely debated. Another was that the role of practitioners’ emotions in noticing and responding to child sexual abuse in a highly gendered workplace was ignored. Did the task of protecting children from sexual harm require only knowledge, thought, and action or did practitioners require an affective self-awareness, emotional resilience, and space for reflection to fulfil the task?

In recent years, historians have considered the interplay of work, gender, and the emotions. Claire Langhamer noted that in the mid twentieth century the “conception of employment as an emotion-free space” began to dissolve, and yet in terms of the professional opportunities available to women, there was a persistent emphasis on “a feminine duty of care whether to children, the sick and vulnerable, or to their male co-workers and bosses.” Her example of a magistrate commenting on “very small children, who have been beaten and neglected by their parents, clinging to the women police and beaming at them lovingly…” is apt here; women were assumed to have “an innate capacity for unremunerated emotional labour.”^12^ Agnes Arnold-Forster has described the pervasive image of doctors, and particularly surgeons, as “emotionally detached” in discourse during the twentieth century.^13^ Sarah Chaney has traced the evolution of the ideal qualities of the nurse which shifted from “sympathy” in the interwar period, a quality associated with patient management, to “compassion” in the early twenty-first century, a term associated with patient satisfaction.^14^ Harry Oosterhuis and Cecile Aan de Stegge have shown that the “proper balance between involvement and detachment” for Dutch mental health nurses shifted over the twentieth century, and increasingly validated their “reflexive thoughtfulness and emotional labour.”^15^ These historians have highlighted that gendered stereotypes of emotional traits or tendencies are tenacious, but equally that they are neither monolithic or timeless, they are constructed and actively maintained or resisted. Still, the tropes of the tactful female nurse who could manage patients’ feelings without being subsumed by her own in order to ensure the directions of the cool, detached doctor were carried out has had a long afterlife despite dramatic shifts in medicine’s gender balance since the 1970s.

Below, the ways in which ideas about child sexual abuse were introduced to Britain by feminists and US medics from the late 1970s are examined. Although the views of UK feminists who came to public attention out of the Rape Crisis movement had some exposure through the media, practitioners were influenced to a greater extent by the awareness-raising activities of psychiatrists and paediatricians. Their reputations as emerging “experts” in child sexual abuse were somewhat tarnished by the critique that arose out of the *Report of the Inquiry into Child Abuse in Cleveland 1987*,^16^ but that commentary also had a negative impact on ordinary health practitioners, engendering a greater fear of “getting it wrong.” It built on the observations of multiple earlier inquiries about their “failures” in child protection. Whether before or after Cleveland, practitioners encountered little useful professional education on child sexual abuse. The courses that did address the emotional aspects of identification were rare and practitioners’ experience of being required to “do more” on the issue came alongside considerable professional jostling as health visitors, GPs, paediatricians, and social work practitioners struggled to establish who would have status as child protection professionals.

Although preventing and responding to harm are multi-agency endeavours that span voluntary and statutory organisations and professional groups, the focus of this article is entirely on health practitioners (community-based nurses and doctors). There is not space here to describe in detail the substantial early NSPCC efforts to raise awareness of all forms of abuse, including sexual molestation. Neither is there scope here to discuss social workers, whose changing roles since the 1960s have been addressed thoroughly elsewhere.^17^ For the purposes of this article, it is important to note the way that the children’s social work delivery model shifted away from broad community engagement to the screening, assessment, and “risk management” of families meeting specific thresholds over recent decades. This has meant that the task of “noticing” signs of child sexual abuse in local families who were not otherwise known to statutory services fell more heavily to those working in the more accessible universal settings of nursery, school, and primary health care. Hence, the emphasis here is on understanding historically when and how health care practitioners were called upon to “notice the signs.” Although not a familiar acronym, community health practitioners are referred to as CHPs below for the sake of brevity.

Archival sources used included trade and professional journals, training materials, government reports, and textbooks. New oral history interviews were undertaken with practitioners who worked in different parts of the UK. Potential participants were contacted via a variety of routes including outreach to professional and child protection organisations, ‘snowball’ sampling and ‘cold contacts’ to practitioners who were identified in trade and academic journals.

## “Not a single mention of child abuse”: professional silences in the 1970s

Although sexual abuse emerged as an area of concern for medics in the US in the 1970s, it was rarely mentioned in the trade press or journals of GPs and health visitors in the UK in that decade. However, social problems such as rising sexual promiscuity, mental illness, and drug use were increasingly debated, and between 1968 and 1978, “baby battering” began to feature in the UK’s *Health Visitor* journal and in the doctors’ trade newspaper *GP*. ^18^ In the same decade, the sexual molestation of children was mentioned only twice in the former publication and not at all in the latter. One of these early references was made in 1976 by a health visitor called Iris Wheway. In a letter to the editor, she described a family on her caseload in the city of Coventry whose four pre-school aged children were “neglected by their poor downtrodden” mother and were being “beaten and *sexually assaulted by their father* [emphasis mine].”^19^ Wheway’s nonchalant tone suggests that for some CHPs encountering incest (as it was then known) was unremarkable, but her explicit naming of sexual abuse was not commonplace at this time.

Most adult survivors who had sought help as children were met with denial. A survivor described that at aged nine (in the 1970s), her mother realised that her stepfather was raping her. Her doctor advised them “to start again in a new home and forget about it, not to speak of it again so that I would forget it.”^20^ Some doctors and nurses did report abuse to the NSPCC, social services or the police, but most did not, whether out of ignorance or trepidation about interfering in private family terrain.^21^

From the mid 1960s, some professional courses for CHPs began to refer to child abuse. In her interview with me, Jennifer recalled that her health visiting course in 1965 in Leeds made no mention of either physical maltreatment or sexual abuse. In contrast, Patricia was taught about the “battered baby syndrome” when she trained as a health visitor in Chiswick in 1967. She was instructed to look out for “any bruising or unexplained incidents.”^22^ Her training also touched on incest which, she was taught, might happen when “the wife had become almost prematurely old and had had several children— was worn out basically and the father might turn to the eldest daughter for sexual needs.” This “definition” was durable and I will return to it below.

Jane Bramwell had a very different experience during her nurse training at Great Ormond Street Hospital for Sick Children (GOSH) in 1966. The lecturer for child psychology shared children’s artwork with her class, explaining that it was created by children who had possibly been abused. Jane said, “I vividly remember it to this day and him showing us pictures of aeroplanes painted in brown … we were sceptical and as youngsters of eighteen from fairly privileged backgrounds … we really didn’t understand what he was trying to get across.” The psychologist was explicit in saying that the brown symbolized the “rectal” and that this particular child’s painting would “lead him to delve further and to have suspicions [of sexual abuse].” Jane remembered the lecture more than fifty years later, musing that, “it must stand out in my mind because it was … quite outside my personal experience in any shape or form.”^23^ Patricia and Jane’s accounts suggested that when sexual abuse within the family was referenced in professional education in the late 1960s, it was introduced in a way that hinted at the influence of psychoanalytical traditions.^24^ Most practitioners in training, however, were told little or nothing about child abuse even well into the following decade. A London-based paediatrician recalled that there was no reference to child protection during her training in the late 1970s.^25^ Similarly, a psychologist remembered that during the two years of her clinical training at Leeds (1978-80), “there was not a single mention of child abuse. Not a word.”^26^

Even though the mainstream press had begun to report on “child sexual abuse” from the mid 1970s,^27^ it did not emerge as an issue that CHPs should pay attention to until the 1980s. Partly, this was because GPs and health visitors were uncertain whether social and “psychosexual” problems should be included in their remit and felt ill equipped to respond “in a subject which figures on hardly any syllabus.”^28^ It also stemmed from the fact that the pressure to respond to violence against women and children came initially from outside of the medical sphere, through the women’s movement and rape crisis activism.

## Feminism and Medicine: clashing concepts of child sexual abuse in the 1980s

In the early 1970s, Rape Crisis Centres and incest survivor groups were established in the UK. Designed for adults who had been sexually assaulted or raped, they had to adapt swiftly when adult survivors spoke of childhood abuse and young girls themselves began to ask for help.^29^ UK feminists emanating from the rape crisis movement played an important role in raising awareness about child sexual abuse across the UK, which they achieved by disseminating information through local networks, generating interest from journalists resulting in articles in the mainstream press, and publishing their own findings.^30^ Their campaigning generated public concern but they had little influence on health professionals.

Doctors preferred to learn from their own. Radiologists and paediatricians had focused attention on parents’ physical abuse of their children in the 1960s, labelling it the “battered baby syndrome.”^31^ From the late 1970s, American physicians such as C. Henry Kempe from Denver and Suzanne Sgroi from Connecticut medicalized the sexual abuse of children in a similar fashion. In the UK, the same pattern unfolded.^32^ Thus the role of UK feminists was occluded. Doctors were credited with discovering child sexual abuse, classified it as a syndrome, and began to control the narrative about how society should respond.^33^

Kempe’s influence was particularly important. In 1978 he lectured in London, where he emphasised the damaging lifelong impact of incest on children.^34^ Later that year, wife and husband Ruth and Henry Kempe (both paediatricians) co-authored *Child Abuse* in which they stated that a mother might collude in the abuse to “hold on to her man for her own needs,” particularly if she was “frigid, rejected sexually, or herself promiscuous.”^35^ British medics followed the Kempes’ conceptual framework. At the 1980 annual meeting of the newly established British Association for the Study and Prevention of Child Abuse and Neglect, the outgoing Chair Dr Alfred White Franklin stated that sexual abuse was “a dysfunction involving all the family members, adolescents being frequently most directly involved.”^36^

Following a lecture by the Kempes at the London headquarters of the CIBA Foundation (an educational and scientific charity funded by a Swiss pharmaceutical company) a study group formed which met at CIBA between September 1981 and March 1984. Dominated by (mostly white male) medics, they produced an influential treatise on sexual abuse.^37^ Like the Kempes, they emphasized a mother’s culpability in the phenomena for “… a man deprived of his conjugal rights may turn to the nearest available source of gratification – a dependent child.”^38^ The medics’ interpretation sounded uncannily like that taught to Patricia on her health visiting course more than a decade earlier and suggested that a patriarchal understanding of women’s role within the family permeated deeply in thinking about sexual abuse. Yet this was wrapped in theoretical sophistication by psychiatrists such as Tilman Furniss in his description of patterns of familial behaviours.^39^

Psychiatrists and medics gave only slight acknowledgement to feminist experiential or theoretical knowledge,^40^ and implied that their own perspective was apolitical. UK-based feminist scholars associated with Rape Crisis activism disagreed, seeing the “dysfunctional family” interpretation as incorporating “the most reactionary sexual politics” in which the extent of “mother blaming” was “quite breathtaking.”^41^ In their view, “the location of power in men/fathers allows them to abuse women and girls in all situations.”^42^ Liz Kelly denounced the “‘expert’ take-over” of child sexual abuse, which caused it to mutate from a political issue “about which feminists have much to offer in terms of theory and practice” to a medicalized matter to be dissected into “‘diagnosis,’ ‘management’ and ‘treatment’ which is the preserve of professionals.”^43^ And while second-wave feminists grappled with the structural barriers that exacerbated the problems faced by abused Black and minoritized women and girls, they criticised the medical establishment for barely acknowledging the added impact of racial stereotyping and racism.^44^

Each vanguard group thought about sexual abuse in a different way. Robert Proctor’s concept of agnotology, how ignorance is used or maintained in a range of settings, is useful here. The medics saw themselves as at the forefront of a crusade to banish ignorance about sexual abuse, believing it to be “a place where *knowledge has not yet penetrated*.” Once a wider swathe of practitioners was educated on the topic, it could be combatted. UK feminists understood ignorance about child sexual abuse as a deliberate and “strategic ploy.” Ignorance about its extent and impact was maintained to bolster the patriarchal power structure.^45^

Feminist theorizing about sexual abuse and its structural and political enablers was portrayed as subjective. Informed by individuals’ “experiences, feelings, beliefs and desires,”^46^ it was suspected of being “biased, prejudiced and partisan.” As Sara Ahmed has remarked, feminists who spoke out against “established ‘truths’ were often “constructed as emotional, as failing the very standards of reason and impartiality that are assumed to form the basis of ‘good’ judgement.”^47^ In fact, Christina Scharff found these tropes about feminists were still in circulation in 2009; across her interviews, feminists were characterised as “angry, aggressive, defensive, making noise, and women who want to ‘fight.’”^48^

In the 1980s, the medics’ perspective was seen as “objective,” appearing to offer a reality unaffected by “the vagaries of human perception, personal interpretation, past experiences and preconceived expectations.”^49^ Although the concept of a neutral “mechanical objectivity” was succeeded in the twentieth century by the notion of trained judgement - the “self-confident expert trusted to judgment informed by well-schooled intuition” - the authority of late twentieth-century doctors and psychiatrists was bolstered by both notions. And as Lorraine Daston and Peter Galison pointed out, “objectivity” remained “powerful as both ideal and practice,” so that ‘objective’ was often used as a synonym for ‘scientific.’” ^50^ The medical/ psychiatric theories were “scientific,” the feminist theorizing was not.

It was the medical conceptual model that CHPs mainly encountered in the workplace. It denied individuals’ “experiences, feelings, beliefs and desires,” and encouraged a mechanical and procedural response to sexual abuse. The emphasis was on checklists, screening, adherence to instructions, binaries of right or wrong in terms of the action to be taken in the event of a disclosure or an indicative sign of sexual abuse. This model disallowed professional or personal anger about the extent of male sexual violence against children. There were hints too at the exclusionary direction of travel. The male-dominated professions of medicine and psychiatry would provide “expert” and “objective” answers to questions about recognition and response; there would be little space for interrogation of the emotional components.

## Awakening CHPs to tackle the “last taboo”

GPs and health visitors perusing their professional magazines in search of interesting research or job opportunities in the early 1980s could not fail to notice articles exhorting them to tackle the “last taboo” of incest. They had “swept it under the carpet,” but must now “be on guard” against it. They were seen as ideally placed to spot the physical or behavioural signs of child sexual abuse or elicit a disclosure.^51^ Early articles were about charitable efforts or short reviews of US publications, but British paediatricians and psychiatrists soon joined the awareness-raising. They spoke at conferences, ran training courses, and published articles targeted at practitioners who worked with children.

The CIBA group had recognised that a community-based doctor or nurse might well be “the first person to recognize” sexual abuse and had set out their “specific responsibilities and tasks.”^52^ However, even as late as 1987, there was little guidance for these practitioners about how to raise their suspicions if a mother or child disclosed abuse or the doctor found signs on examination.^53^ GOSH psychiatrist Eileen Vizard warned that if health visitors and GPs waited for clearer national guidelines, “many young children [would] continue to be sexually abused, often within their own family circles.” They must respond “in a serious and concentrated way,” and “bring many *more* cases of sexual abuse to public notice and hence stimulate further pressure for guidelines.” By intervening early, they could also be a part of preventing later mental illness and maladjustment.^54^”

At Leeds General Infirmary in the early 1980s, paediatrician Michael Buchanan established a child abuse team with paediatricians Chris Hobbs and Jane Wynne and psychologist Helga Hanks. The Leeds team is remembered today mainly in relation to their use of anal dilation for diagnosis, which would become highly controversial in Cleveland. Their finding that the anal rape of very young children was “a serious, common, and under-reported type of child abuse,”^55^ deconstructing White Franklin’s myth of the adolescent incest victim, was also pivotal and highly relevant to the work of CHPs. However, they played a wider role in the 1980s: Hobbs and Wynne gave talks all over the country, wrote many articles, produced videos, and ran training courses. Writing about the management of sexual abuse, Hobbs and Wynne commented that “few doctors would be able to talk about sexual abuse without feeling upset.”^56^ In a 1989 newsletter for community paediatricians, they explained that GPs had as much personal experience of child abuse as found in the general population, and that “painful forgotten experiences may affect an individual’s capacity to become involved in child sexual abuse work.”^57^ Quite what a doctor should do with these feelings was not addressed.

The Great Ormond Street Hospital sexual abuse team established by Bentovim in 1981 assessed and treated children following a medical examination elsewhere. They regarded consultation and teaching of hospital and community-based medics and other professionals as one of the team’s main functions.^58^ Paediatricians and psychologists were invited into the hospital to observe their work or co-deliver therapeutic groups. The team also worked in primary care settings to increase awareness. For example, Furniss facilitated a multi-disciplinary case discussion group at a north London GP practice. ^59^ Vizard ran a reflective group for north London health visitors and lobbied for training to help health visitors and GPs to be “alert to the existence of CSA.”^60^

The Leeds and GOSH teams were at the vanguard in creating an awakening in relation to sexual abuse in primary care. They expended considerable energy in education and outreach. They demonstrated some awareness of the effects of practitioners’ own emotions in enabling a productive response to child sexual abuse. However, as the medical and psychiatric model was disseminated across the country, that acknowledgement of practitioners’ feelings was lost. The task became to spread “factual” knowledge about the signs of abuse and what procedures to follow. It became a technical mission. A practitioner’s emotional reactions or sensitivities to the complexities of relationships, sexual practices, coercion, power and authority, secrecy and shame were rarely considered.

## Training in the 1980s: “Almost too difficult for us to do in any meaningful way”

Although awareness raising in the broad sense proliferated, there was a dearth of formalised training available on sexual abuse in the 1980s. Short introductory packs for trainers could be purchased from the NSPCC.^61^ The University of Leeds made two video packs available for mail order covering definitions, impact, procedures, investigations, therapy and “Talking with Sexually Abused Children.”^62^ The CIBA Study Group continued to work together as the Training Advisory Group for Sexually Abused Children and disseminated information on training courses and materials to the range of professions working with children.

The curriculum for health visitors and GPs coming into their professions still barely touched on child sexual abuse. A Manchester based health visitor recalled that there were “definitely slots for this but it was treated I— looking back on it now— as far as I remember, it was treated as a kind of separate thing.” She remembered being shown a video about child abuse, “the lights went down and— It was almost like something we had to do and it was important but it was almost too difficult for us to do somehow in any meaningful way.”^63^ As social work academic Olive Stevenson pointed out in 1989, professional training for those involved in child protection “foster[ed] the denial of [emotional] involvement rather than its acknowledgement and constructive use.”^64^ Once practitioners were “in service,” the general direction of travel was towards multi-disciplinary child abuse training led by Area Review Committees (ACPCs). These had been established following the murder of seven-year-old Maria Colwell in 1973 by her stepfather; the subsequent inquiry found failures in inter-agency communication and procedural lapses. ARCS (renamed Area Child Protection Committees (ACPCs) in 1980) were therefore introduced to co-ordinate the agencies responsible for responding to children at risk of harm. Their interdisciplinary training and that of employers providing short single agency courses had a heavy emphasis on procedural compliance and the practical actions to be undertaken once abuse had been identified, rather than on early recognition or emotional engagement with children and parents.

Although the Open University’s Child Abuse training pack of 1978 made no mention of sexual abuse,^65^ the course it developed jointly with Newcastle upon Tyne Polytechnic a decade later addressed sexual abuse throughout.^66^ ‘Child Abuse: A Teaching Pack’ was funded by the Department of Health and Social Welfare; the course included exercises, case studies, and a guide for teachers. Although suitable for any student with ‘an interest in the subject,’ it was promoted as a valuable resource for ‘professional workers in the field’ and designed with the expectation that many of its participants would be studying as part of a group. Its authors claimed that the course’s “most unusual and striking characteristic” was “its acknowledgement of and focus upon personal feelings and the emotional impact of the subject matter on participants.” The social workers, parents, education, and health practitioners who were part of eleven groups that piloted the materials in the year prior to publication advised that it was “only by facing and working though their own feelings and memories that they were able to ‘get on top of’ the subject matter, and gain the self-confidence to deal with it.” There was distinct ambivalence about privileging affect, however, and the authors verged on the apologetic about making reference to emotions, noting they felt they should not place “too much emphasis on this area” but must not deny the “distress and discomfort” that the subject could engender.^67^

Within the teaching pack, a series of student workbooks encouraged participants to consider their feelings and thoughts about situations (“feel, think, do”) before deciding on any possible action. The importance of seeking their own support system whilst studying was emphasised, perhaps undertaking the course or activities with a partner, colleague or friend. Sexual abuse was integrated into each course component ranging from a fictitious case study through various experiential activities. For example, in an introductory activity, one scenario described six-year-old Tracy, whose father had been grooming her. Over time, he encouraged her to participate in more overtly sexual acts after which “he kissed Tracy lovingly, told her she was a good girl and that he loved her and that this was her special way of showing him that she loved him.”^68^ Such examples helped to encourage students to get a sense of the loyalty and love children often felt towards their abuser; and why children might find it hard to understand that the abuse was wrong or to tell anyone about it.

By telling students that those who had piloted the course reported feeling distress, pain and guilt, permission was granted to acknowledge their own feelings. For the pilot groups, the distress arose from their own memories of abuse and betrayal by trusted adults. Their own treatment of children “including, for some people, painful recognitions that they had themselves been abusers” provoked guilt. They might experience embarrassment and fear about their reactions as adults. Perhaps they had responded “clumsily or in ignorance to delicate situations which involved reporting abuse” or were frightened to take the risk in “making such terrible decisions with such far-reaching consequences, often on very little information.”^69^

Mentioning in the materials that “some people” might recognize themselves as abusers was radical. This Open University (OU) course was also atypical in the way it provided social workers, health practitioners, and others with a (limited) space to think about the motivations of perpetrators and the experiences of survivors. They could listen to audio of interviews with Tom, Michael, and George, three men serving sentences for child abuse at Grendon, a therapeutic community prison, and consider whether abusers should be treated or punished.^70^ Survivor Richard Johnson’s audio interview described his father’s sexual abuse of him and his siblings, his experience of the investigation, and the long-lasting effects of both on his life.^71^

Through experiential learning, accounts of lived experience and academically informed literature, students who participated in groups that used this OU training pack had a rare opportunity to think about the impact of their own feelings in taking on a more proactive approach to abuse. How would their own past experiences of family, sexual relationships, violence or coercion affect their emotional responses to children and their ability to interpret and intervene? In relation to physical abuse and neglect, governmental and media judgements about their actions or failures to protect children had been harsh over recent years. To give just one of many possible examples, following the death of Kimberley Carlile (1986), the family’s health visitor was criticised for becoming “infected with the occupational disease of drift and inaction.”^72^ What would be the future emotional costs for GPs or health visitors in responding to sexual abuse with its additional layers of secrecy and denial?

The OU course was exceptional in terms of its attention to subjectivity and the role of the emotions. Most CHPs did not have access to courses of this nature as part of their professional development. Furthermore, no matter how sensitively curated and delivered a training course was, it could only begin the process of building practitioners’ confidence. To be effective, there had to be space for contemplation in the workplace. A minority of CHPs had access to a reflective space such as those set up and run by Furniss and Vizard in north London for a brief period. Another example was Balint seminars, established in the 1950s to provide a reflective forum for a small minority of GPs. ^73^ Typically, a case presentation preceded a discussion which emphasized the interpersonal and emotional content of interactions with patients. Multi-disciplinary seminars were established in some of the more forward-thinking health centres and group practices. These opportunities were rare, however, and often short-lived. Most CHPs returned to a professional environment which did not provide a forum for discussion of personal responses or emotional expression.

## Mistakes and rivalries in the late 1980s

As the 1980s drew to a close, the dialogue about sexual abuse became increasingly fractious and there were misgivings voiced about “experts.” Most accounts relate this scepticism to events in Cleveland in northeastern England between March and July 1987, when the numbers of children suspected of having been sexually abused rose very suddenly. Over a period of five months, 121 children were diagnosed by two local paediatricians and admitted to Middlesborough Hospital.^74^ Much was made of the fact that 98 of the 125 children diagnosed had been returned home by July 1988 and proceedings in 27 wardship cases had been dismissed.^75^ Behind the scenes, however, an adviser warned the Chief Secretary to the Treasury that the “DHSS have told us that an independent medical assessment has been made and that the diagnoses of sexual abuse were correct in at least 80% of the 121 cases.”^76^

Serious rifts had developed in Cleveland during the crisis, with social services and health personnel at loggerheads with the police. The government ordered an independent inquiry, which found that the children’s needs had been obscured by failures in co-operation and communication between the services.^77^ The scale of the removals, the sense that paediatricians, health visitors and social workers interfered too swiftly, and without due regard for parents’ rights provoked strong reactions from politicians and the press.

Much of the media outrage focused on the paediatricians’ reliance on the anal dilation test described by Hobbs and Wynne in their 1986 article in *The Lancet*. A slew of letters to the editor followed its publication, criticising both the Leeds diagnoses and the use of anatomically correct dolls for interviewing children. The most extreme reaction came from Elizabeth Tylden who railed against the “medical rape” of children. She described a seven-year-old girl who “violently resisted inspection of her bottom” by a woman doctor; was “subjected to a session with ‘dirty dolls’” where “voyeurs behind a screen” created “a Kafka like setting for the perversion of innocence.”^78^ There was a deep unease about looking for the physical signs of sexual molestation and talking to children about sexual activities as breaking up “a previously happy family, on circumstantial evidence that proves to be misleading, is to initiate disaster.”^79^ Some took a less sensationalist stance, challenging the validity of the research findings, positing other possible explanations and urging further careful research.^80^ The strength of the physicians’ emotional responses foreshadowed much of the reaction to Cleveland.

Publicity about Cleveland led to “the prioritization of law-and-order solutions for child abuse, rather than trusting in the expertise of psychiatrists, social work professionals and medical practitioners.”^81^ Doctors were in a dilemma; they warned that “failure to recognise the problem can lead to continuing severe and unnecessary distress in the child” but that “the diagnosis must be made and action taken against the knowledge that a mistaken diagnosis can be destructive to future child and family happiness.” ^82^ Procedures were introduced to promote consistency in investigations and evidence gathering. ^83^ The earlier ideological battles about how practitioners should understand child sexual abuse between feminists and medics/ psychiatrists were less evident as a more forensic approach took hold.

And yet even as trust in the experts waned in the aftermath of Cleveland, there was a consolidation of the notion that this was a frontier so perilous that only “experts” could navigate it. The public and press did not always distinguish between primary care health practitioners and specialists, which left some CHPs feeling as criticised as the “experts.” Cleveland was the first major sexual abuse scandal in the UK and certain aspects of the inquiry’s findings had not been articulated before. But in other ways, it merely amplified a litany of problems that had been rehearsed for a decade or more. Nearly a score of child abuse inquiries had criticised professional behaviours.^84^ The notion that interprofessional fractures arose as much out of subjectivity and differing emotional responses to abuse as out of procedural failings was not entertained.

In fact, deep seated rivalries ran between the professional groups involved in protecting children in this period. For example, some health visitors felt displaced and undervalued when social services departments were established in the early 1970s.^85^ Health visiting existed as a profession since the late nineteenth century with the aim of combatting infant mortality, mainly through maternal education in the home. The role expanded beyond babies to the prevention of ill-health for the wider family when the NHS was set up in 1948. Some health visitors maintained that holistic role into the 1970s although increasingly they were redirected to new births and pre-schoolers.^86^ Their role in relation to child welfare was bitterly disputed within the profession itself, however, with some adamant that health visitors were not “primarily concerned with needy people, or people in distress, but with people living their ordinary everyday lives,”^87^ whilst others wanted “recognition of the social work content of our job.”^88^ *Health Visitor* editorialised that "despite murmurings that no one profession is trying to stake a claim in this kind of work [child welfare], in practice the different professionals end up at loggerheads.”^89^ This discomfort about being “uncomfortably sandwiched between nursing and social services…” went back decades and continued throughout the 1980s.^90^

The relationship between health visiting and general practice was also challenging. Writing in 1989, Stevenson was one of the few contemporary commentators to untangle the emotional cords wrapped around practitioners in relation to sexual abuse. Sexism made it difficult for women to interject into discussions and, when they did, their views were dismissed. Although health visiting had more radical and assertive roots than nursing, both had lower status as female-dominated professions.^91^ Health visitors formed an “extensive bridge between frontline agencies and the core professions” of the police, social work, and paediatricians,^92^ but they were often silenced by doctors, many of whom perceived themselves as “top dogs” trained to “exercise authority” even in “matters which have little to do with medicine.”^93^ Although many more women were training as GPs, they were often in part-time junior positions once qualified, in effect subservient to male colleagues in a practice.

Consideration of possible abuse at case conferences rarely explored the “feeling component … with any openness.” Where there was a possibility of child sexual abuse, these barriers were compounded by “mechanisms of denial,” sometimes compounded by an “element of voyeurism, even excitement … probably accompanied by guilt.” Feelings about “gender roles and male oppression” lurked just beneath the surface of such discussions. The physical “signs” were often inconclusive or absent therefore practitioners had to rely on “soft” information, which was necessarily subjective and affected by “powerful feelings about what is best for children.”^94^ Gender shaped practitioners’ responses and their perceptions about who might recognise or deny sexual abuse. When health visitor Wheway, referred to above, “named” sexual abuse in one of “her” families, it was in support of her argument that (female) health visitors should retain independent status rather than come under the control of the local (usually at that time male) GP. She argued that professional autonomy enabled health visitors to gain privileged access to families within their homes and to build trust in order that families could reveal intimate details of their lives.

Most CHPs had not embraced the recognition of sexual abuse as a routine part of their practice by the time Cleveland was in the news. They were held back by a lack of training, emotional factors that affected their own practice and relationships but were almost never acknowledged, and a lack of professional regard for their contribution. Theirs was a fledgling interest without the “muscle” that resources and reflective practice spaces would have provided to support it to take flight.

## Conclusion

Cleveland is perceived as a watershed moment after which practitioners retreated behind a wall of procedures and protocols, having found the potential professional and emotional costs of identifying and responding to sexual abuse too great. “[I]nvestigation, culpability and potential prosecution” became the leading preoccupation,^95^ undermining efforts to encourage those in universal settings like health centres to be confident in recognizing the alerting signs. Although “expert” credibility came under concerted attack, the necessity for “experts” was not ultimately contested and, after all, those specialists who were confident enough to remain in the field under fire had even more status in the long run. They took up university professorships, advised government departments, and continued to publish widely.

A decade earlier, the vanguard “experts” had recognized CHPs as having the skills and opportunities to identify children who were being sexually abused. That role in recognition was subsequently incorporated into guidance. Overall, their function in relation to sexual abuse appeared to the newly constituted “experts” and to the government as “commonsense” and unproblematic. As I have shown above, it was not. Although different interpretations of the causes and meanings of child sexual abuse emanated from feminist activists and medics, it was the latter perspective that found purchase in the health journals and local sites of healthcare delivery. This conceptual model paid little attention to the feelings that might be evoked in staff who were expected to “notice” sexual abuse or the need for them to have safe spaces for individual and group reflection. Few doctors or nurses received formal training in detecting and responding to child sexual abuse and professional curricula were slow to respond to these new expectations.

Other factors hindered the ability of CHPs to adapt their professional practice to recognize the early signs of sexual abuse. Although the gender imbalance in medicine, psychiatry and general management roles had begun to shift, senior positions in clinics, GP practices and case conferences were dominated by men. The complex emotional components of sexual abuse, the need for reflexivity and structures of support were ignored. The views of those seeking to voice supposedly “softer” perceptions or suspicions about sexual abuse were often dismissed or trivialized.

Children have borne the brunt of the failure to fulfil the potential of the CHP role in sexual abuse. Even as the OU course described above created a space to acknowledge practitioners’ emotional labour, it echoed the Cleveland Inquiry in restricting the role of practitioners in terms of who could talk to children about sexual abuse. Students were warned about “disclosure,” a term that had come to denote specific processes in relation to the investigation of sexual abuse that were now the preserve of specialists. Non-specialists should “*limit* how much of a ‘disclosure’ the child makes.” They apparently lacked the technical skills (e.g., in using play, art or anatomically correct dolls) to elicit reliable information, leaving the child’s evidence open to challenge in court. If a child spoke about “something that is distressing or bothering them,” the adult should not be “*actively* trying to persuade them to give … a detailed ‘disclosure’ of what has happened.” They must be wary of leading the child and must ensure “that the conversation does not progress further than you (or the child) can comfortably cope with,” before finding the “right people to ‘disclose’ to if necessary…"^96^ The “right people” might be “experts” in technique but they would, of course, usually be complete strangers to the child, making the likelihood of the child trusting them very low indeed. This policy not only devalued the skills of community practitioners but reduced the likelihood that the child could speak and be heard, for as Margaret Rustin noted “[w]ithout the involvement of teachers or others in the community who know a child over time, the professional task of child protection is up against exceptional odds.”^97^

This was a far cry from the original thinking of the multidisciplinary teams at Leeds and Great Ormond Street, or the UK feminists who came out of Rape Crisis Centre activism. For all their theoretical disagreements, they agreed on the need to listen and respond to children. Although well-intentioned, the consequence of the later restrictive guidance was to hinder adult/child dialogue and provoke professional anxiety; this was emphasized by many of the practitioners I interviewed decades later. A consultant community paediatrician described the fear of “getting it wrong”:

… People are really, really terrified about asking children if they are being sexually abused. The [school] nurse isn’t going to say to that child who comes in with tummy ache, ‘and has anybody done anything you don’t like…’ Because the police would say ‘you put ideas into their head, didn’t you?’ … [W]ith GPs, if a child comes in with abdominal pain, recurrent urinary tract infections, vulval vaginitis, they’re not going to ask the child. They’re terrified.^98^

In the 1990s, structural changes further fettered community health practitioners’ capacity to respond to child sexual abuse. A governmental obsession with demonstrating efficiency diverted CHPs away from relationship-based practice and toward increasingly routinised and centrally controlled activity. A growing trend of managerialism introduced the watchwords of improvement, quality of care, and performance management.^99^ As a consequence of shrinking public investment and the political drive towards a “competitive market” health economy, the bureaucratic demands on CHPs expanded leaving less time available for practitioners to develop trusting relationships with families. As a Manchester-based health visitor recalled, “at that time [it was] about counting, it was all about how many of something you did. So we had these little machines where you had to count all your contacts every week.’^100^ The emphasis was not on the quality of the relationships with families or what was noticed on a baby’s body or disclosed by a concerned mother, but on the number of families seen or the percentage of babies immunized in each area. This direction of travel continued in the decades that followed. It was antithetical to the sort of environment that would promote the early identification of sexual abuse within the family or indeed of other forms of abuse, but its impact has barely generated discussion. The great potential of family doctors and health visitors to be allies of the sexually abused child, through being alert to “every signal that an abused child gives out” has not been realized.

## Footnotes

1 “Childhood Matters: Report of the National Commission of Inquiry into the Prevention of Child Abuse,” (Great Britain: Department of Health, 1996), 50.

2 Letter from female survivor (age unknown) to The National Commission of Inquiry into the Prevention of Child Abuse (NCIPCA), 6 March 1995.

3 Letter from female survivor (age approximately twenty) to NCIPCA, 1995.

4 *Child Protection: Guidance for Senior Nurses, Health Visitors and Midwives* (London: HMSO, 1988); *Diagnosis of Child Sexual Abuse: Guidance for Doctors*, (London: HMSO, 1988); “Working Together for the Protection of Children from Abuse,” (London: DHSS, 1988).

5 “Childhood Matters,” 172.

6 Liz Kelly and Kairika Karsna, “Measuring the Scale and Changing Nature of Child Sexual Abuse and Child Sexual Exploitation - Scoping Report,” (London: London Metropolitan University, 2017), 18.

7 Kathleen Coulborn Faller, “The Child Sexual Abuse Disclosure Controversy: New Perspectives on an Abiding Problem,” *Child Abuse and Neglect* 99 (2020) 104285, 1-7; Debra Allnock and Pam Miller, “No One Noticed, No One Heard : A Study of Disclosures of Childhood Abuse,” (London: NSPCC, 2013), 23.

8 George Behlmer, *Child Abuse and Moral Reform in England, 1870-1908* (Stanford, Ca: Stanford University Press, 1983); Roger Davidson, “‘This Pernicious Delusion’: Law, Medicine, and Child Sexual Abuse in Early-Twentieth-Century Scotland,” *Journal of the History of Sexuality* 10 (2001): 62-77; Lucy Delap, “‘Disgusting Details Which Are Best Forgotten'”: Disclosures of Child Sexual Abuse in Twentieth-Century Britain," *Journal of British Studies* 57, 1 (2018): 79-107; Linda Gordon, *Heroes of Their Own Lives: The Politics and History of Family Violence Boston, 1880-1960* (London: Virago, 1989); Carol-Ann Hooper, “Child Sexual Abuse and the Regulation of Women: Variations on a Theme,” in *Gender Violence: Interdisciplinary Perspectives,* ed. Laura L. O’Toole, Jessica R. Schiffman, and Marge L. Kiter Edwards (New York: New York University Press, 2007), 53-77; Louise A. Jackson, *Child Sexual Abuse in Victorian England* (London: Routledge, 2000); Stephen Robertson, *Crimes against Children : Sexual Violence and Legal Culture in New York City, 1880-1960* (Chapel Hill: University of North Carolina Press, 2005); Lynn Sacco, *Unspeakable : Father-Daughter Incest in American History* (Baltimore: Johns Hopkins University Press, 2009), 214.

9 Steven Angelides, *The Fear of Child Sexuality: Young People, Sex, and Agency* (Chicago: The University of Chicago Press, 2019); Harry D. Hendrick, *Child Welfare: England: 1872-1989* (London: Routledge, 1994); James R. Kincaid, *Erotic Innocence: The Culture of Child Molesting* (Durham: Duke University Press, 1998).

10 Mical Raz, *Abusive Policies: How the American Child Welfare System Lost its Way* (Chapel Hill: The University of North Carolina Press, 2020), 118, 92-116.

11 “The danger of labelling parents guilty until proved innocent,” *The Independent*, 30 June 1987; Lesley Garner, “Overboard on Child Abuse,” *Daily Telegraph,* 1 July 1987; Mary Riddell, “Salem Comes to Portsmouth,” *The Guardian*, 13 August 2000; Michael Wright, “When Fear Stalks the Innocent,” *The Guardian*, 29 June 1987.

12 Claire Langhamer, “Feeling, Women and Work in the Long 1950s,” *Women’s History Review* 26, 1 (2017): 12.

13 Agnes Arnold-Forster, “Racing Pulses: Gender, Professionalism and Health Care in Medical Romance Fiction,” *History Workshop Journal* 91 (2021): 165.

14 Sarah Chaney, “Before Compassion: Sympathy, Tact and the History of the Ideal Nurse,” *Medical Humanities* 47 (2020): 475-484.

15 Harry Ososterhuis and Cecile Aan de Stegge, “Between Emotional Involvement and Professional Detachment: The Challenges of Nursing in Dutch Mental Institutions (1880–1980),” *Social History of Medicine* 34, 4 (2021): 1278, 1295.

16 Elizabeth Butler-Sloss, “Report of the Inquiry into Child Abuse in Cleveland 1987,” (London: HMSO, 1988).

17 Nigel Parton, *Governing the Family: Child Care, Child Protection and the State* (Hampshire: Palgrave, 1991); *The Politics of Child Protection: Contemporary Developments and Future Directions* (Basingstoke: Palgrave Macmillan, 2014).

18 “New directions in venereal disease,” *GP*, (4 September 1970): 3; “Is drug abuse a symptom of emotional delinquency?” *GP*, (19 January 1973): 18; “Is the British girl more permissive?” *GP*, (1 June 1973): 17.

19 “Letters to the Editor,” *Health Visitor* 49, 12 (December 1976): 411-412.

20 Letter from female survivor (age twenty five) to NCIPCA, 1 November 1994.

21 “Battered Babies,” *Health Visitor*, 46, 7 (July 1973): 243.

22 Oral history interview with Patricia Scowen by author, 19 September 2019.

23 Oral history interview with Elizabeth Jane Bramwell by author, 4 July 2019.

24 Carol Smart, “Reconsidering the Recent History of Child Sexual Abuse,” *Journal of Social Policy* 29, 1 (2000): 66-67.

25 Oral history interview with anon. consultant paediatrician by author, 31 May 2019.

26 Background interview with anon. clinical psychologist, 24 September 2019.

27 Adrian Bingham, “‘It Would be Better for the Newspapers to Call a Spade a Spade’: the British Press and Child Sexual Abuse, c. 1918–90,” *History Workshop Journal* 88, (2019): 90.

28 “The need for psychosexual counselling, *GP*, (5 January 1973): 15.

29 Mary MacLeod and Esther Saraga, “Abuse of Trust,” *Journal of Social Work Practice* 3, 1 (1987): 71.

30 Sarah Nelson, *Incest : Fact and Myth* (Edinburgh: Stramullion, 1982; repr, 1987); London Rape Crisis Centre, *Sexual Violence: The Reality for Women*; Marlene T. Bogle, “Brixton Black Women’s Centre: Organizing on Child Sexual Abuse,” *Feminist Review*, 28 (1988): 132-135; Scholarship on the abuse of boys or the role of women as perpetrators was scarce until the late 1990s, see Jacqui Saradjian, *Women Who Sexually Abuse Children: From Research to Clinical Practice* (Chichester: John Wiley and Sons, 1996).

31 Jennifer Crane, *Child Protection in England, 1960-2000: Expertise, Experience, and Emotion* (2018), 27-44.

32 Ray Eugene Helfer and C. Henry Kempe, eds, *Child Abuse and Neglect: The Family and the Community* (Cambridge, Mass: Ballinger, 1976); Ruth S. Kempe and C. Henry Kempe, *The Common Secret: Sexual Abuse of Children and Adolescents* (New York: W H Freeman, 1984); Suzanne M. Sgroi, ed. *Handbook of Clinical Intervention in Child Sexual Abuse* (Lexington, MA: Lexington Books, 1982); A. Bentovim and M. Lynch, “Recognition and Reporting of Sexual Abuse,” *Archives of Disease in Childhood* 56, 7 (1981): 570; Patricia J. Mrazek, Margaret A. Lynch, and Arnon Bentovim, “Sexual Abuse of Children in the United Kingdom,” *Child Abuse and Neglect* 7 (1983): 147-153; T. Furniss, L. Bingley-Miller, and A. Bentovim, “Therapeutic Approach to Sexual Abuse,” *Archives of Disease in Childhood* 59, 9 (1984): 865-870; Eileen Vizard and Arnon Bentovim, “Incest - the Role of the General Practitioner,” *Maternal and Child Health* (1985): 55-59.

33 Historians have demonstrated that sexually violent acts against children were discussed in medical literature in prior decades, see for example, Louise A. Jackson, *Child Sexual Abuse in Victorian England* (London: Routledge, 2000); Smart, “Reconsidering the Recent History of Child Sexual Abuse, 1910-1960;” “A History of Ambivalence and Conflict in the Discursive Construction of the ‘Child Victim’ of Sexual Abuse,” *Social and Legal Studies* 8, 3 (1999).

34 C. Henry Kempe, “Recent Developments in the Field of Child Abuse,” *Child Abuse and Neglect* 2, 4 (1978): 262-264.

35 Ruth S. Kempe and C. Henry Kempe, *Child Abuse*, (London: Fontana/ Open Books, 1978), 66.

36 Alfred White Franklin, “British Association for the Study and Prevention of Child Abuse and Neglect,” *Child Abuse and Neglect* 5 (1981): 69-70.

37 CIBA Foundation, *Child Sexual Abuse within the Family* (Cambridge: Tavistock Publications, 1984), xii.

38 Ibid, 28.

39 Ibid, 12-13.

40 Arnon Bentovim and Vizard, Eileen “Chapter 1: sexual abuse, sexuality and childhood” in Arnon Bentovim et al, eds, *Child Sexual Abuse within the Family: Assessment and Treatment: The Work of the Great Ormond Street Sexual Abuse Team* (London: Wright, 1988), 1.

41 “Child Sexual Abuse: Towards a Feminist Professional Practice,” (London, 1988), 6.

42 London Rape Crisis Centre, *Sexual Violence : The Reality for Women* (London: Women’s Press, 1984), 89.

43 Kelly, Liz, “Cleveland - Feminism and the media war,” *Spare Rib*, 193 (August 1988): 8-11.

44 Emily Driver and Audrey Droisen, *Child Sexual Abuse: Feminist Perspectives* (Macmillan, 1989), 33, 158-169.

45 Robert N. Proctor and Londa Schiebinger, *Agnotology: The Making and Unmaking of. Ignorance* (California: Stanford University Press, 2008), 11, 15.

46 Jessica Yakeley et al, “Psychiatry, Subjectivity and Emotion - Deepening the Medical Model,” *Psychiatric Bulletin* 38, 3 (2014): 98.

47 Sara Ahmed, *The Cultural Politics of Emotion* (Edinburgh: Edinburgh University Press, 2004), 170.

48 Christina Scharff, “Young Women’s Dis-Identification with Feminism: Negotiating Heteronormativity, Neoliberalism and Difference,” (PhD diss, London School of Economics, 2009), 120, 149.

49 Ibid, 98.

50 Lorraine Daston and Peter Galison, *Objectivity* (New York: Zone Books, 2018), 363, 372.

51 Tom Smith, “Child sexual abuse swept under the carpet,” *Pulse*, 21 February 1981, 54; Tony Whitehead, “Dealing with incest and its aftermath, *Pulse*, 7 May 1983, 68; Andrew Lycett, “GPs urged to be on guard against child sexual abuse,” *Pulse*, 6 August 1983, 10; Jean Moore, “The last taboo: child sexual abuse – its causes and effects,” *Health Visitor*, 54.7 (July 1981): 279-281; Rosalind Meek and Gabrielle Markes, “Sexual abuse of children,” *Health Visitor*, 55.7 (July 1982): 366; Eileen Vizard, “The sexual abuse of children,” *Health Visitor*, 57.8 (August 1984): 234-236.

52 CIBA Foundation, *Child Sexual Abuse within the Family*, 94-96

53 J. M. Leventhal et al, “What to Ask When Sexual Abuse Is Suspected,” *Archives of Disease in Childhood* 62, 11 (1987): 1188-1193.

54 Eileen Vizard, “The sexual abuse of children - Part 2,” *Health Visitor*, 57.9 (September 1984): 279-280.

55 Christopher J Hobbs and Jane M Wynne, “Buggery in Childhood—a Common Syndrome of Child Abuse,” *The Lancet* 328, 8510 (1986): 792-796; “Child Sexual Abuse—an Increasing Rate of Diagnosis,” *The Lancet* 330, 8563 (1987): 837-841.

56 C. J. Hobbs and J. M. Wynne, “Management of Sexual Abuse,” *Archives of Disease in Childhood* 62, 11 (1987): 1184.

57 Christopher Hobbs and Jane Wynne, *Community Paediatric Group Newsletter*, Autumn 1989, 7.

58 Furniss, Bingley-Miller, and Bentovim, “Therapeutic Approach to Sexual Abuse.”, 865-870.

59 Background interview with GP by author, 27 May 2019.

60 Vizard, “The sexual abuse of children - Part 2”: 279-280.

61 Child Sexual Abuse Training Pack, NSPCC (London, 1987).

62 “The Sexual Abuse of Children” and “Talking with Sexually Abused Children,” Leeds University (Leeds, undated).

63 Oral history interview with Jane Hutcheson by author, 4 July 2019.

64 Olive Stevenson, ed. *Child Abuse: Public Policy and Professional Practice* (Herts: Harvester Wheatsheaf, 1989), 187.

65 R. Fothergill and S. Hurley, eds, *Child Abuse – A Teaching Pack* (Milton Keynes: Open University, 1978).

66 *Child Abuse and Neglect: An Introduction*, (P554) (Milton Keynes: Open University, 1989).

67 P554 Course introduction and study guide, 4-9.

68 P554 Workbook 1 Making sense of child abuse, 10.

69 P554 Workbook 2 Recognising and responding, 4.

70 Mark Morris, *Dangerous and Severe – Process, Programme and Person: Grendon’s Work* (London: Jessica Kingsley Publishers, 2004).

71 P554 Audio 2.

72 *Child Abuse: A Study of Inquiry Reports 1980-1989* (London: HMSO, 1991), 83.

73 Balint M. et al, *A Study of Doctors* (London: Tavistock Publications, 1966).

74 Nigel Parton, *Governing the Family: Child Care, Child Protection and the State* (Hampshire: Palgrave, 1991), 79.

75 “Summary of the Cleveland Inquiry,” *BMJ* 298 (16 July 1988): 190-191.

76 R. B. Saunders, memo to John Major, Chief Secretary to the Treasury, 5 July 1988 (The National Archives HPSS T632/139. Cleveland Enquiry into Child Abuse Documents).

77 Butler-Sloss, “Report of the Inquiry into Child Abuse in Cleveland 1987.”

78 Elizabeth Tylden, letter to the editor, *The Lancet,* 31 October 1987: 1017.

79 John A. Davis, letter to the editor, *The Lancet*, 21 November 1987: 1217.

80 H. B. Keen, letter to the editor, *The Lancet*, 31 October 1987: 1018; George Russell, 12 December 1987: 1397; R. Sunderland, 31 October 1987: 1018; B. L. Priestley, 12 December 1987: 1396; J. K. H. Wales and L. S. Taitz, 12 December 1987: 1396-1397; C. M. Ridley, 31 October 1987: 1017-1018.

81 Bingham, “‘It Would Be Better for the Newspapers to Call a Spade a Spade,’” 104.

82 *Diagnosis of Child Sexual Abuse: Guidance for Doctors* (London: HMSO, 1988), 2.

83 *Physical Signs of Sexual Abuse* (Royal College of Physicians of London, 1991), 2.

84 *Child Abuse: A Study of Inquiry Reports 1973-1981* (London: HMSO, 1982), 11-14.

85 “The Seebohm Report.” *Health Visitor*, 42, 1 (January 1969): 7.

86 Jennifer Smith, “Illustrations from the Wellcome Institute Library: The Archive of the Health Visitors’ Association in the Contemporary Medical Archives Centre,” *Medical History* 39 (1995): 358-360.

87 ‘Letters to the editor,’ *Health Visitor* 41, 2 (February 1968): 89.

88 “Definitions,” *Health Visitor* 47, 8 (August 1974): 253.

89 “Books, Books, Book: ‘The Ealing Battered Baby Conference,’” *Health Visitor* 48, 7 (July 1975): 273.

90 Jane Brooks, “‘Visiting Rights Only’ : The Early Experience of Nursing in Higher Education, 1918-1960” (PhD diss, London School of Hygiene and Tropical Medicine, 2005), 278-280.

91 Robert W. J. Dingwall, “Collectivism, Regionalism and Feminism: Health Visiting and British Social Policy 1850-1975,” *Journal of Social Policy* 6, 3 (1977), 315.

92 Elizabeth Birchall, “Working Together? A Survey of Professional Perceptions in Child Protection in England,” (PhD diss, University of Stirling, 1992), 93, 129.

93 Stevenson, *Child Abuse: Public Policy and Professional Practice*, 185-187.

94 Ibid, 194-197.

95 Nigel Parton and Corinne Wattam, eds, *Child Sexual Abuse: Responding to the Experiences of Children*, (Chichester: Wiley, 1999), 6.

96 P554. Workbook 2, 16-20.

97 Margaret Rustin, “Conceptual Analysis of Critical Moments in Victoria Climbié’s Life,” *Child and Family Social Work* 10 (2005), 16.

98 Oral history interview with anon. consultant paediatrician by author, 31 May 2019.

99 R. Griffiths, “Seven Years of Progress: General Management in the NHS,” (London: Audit Commission, 1991); D. M. Berwick, Enthoven A., and Bunker J. B, “Quality Management in the NHS: The Doctor’s Role - II,” *BMJ* (1992): 304-308; “The New NHS. Modern. Dependable,” (London: The Department of Health, 1997).

100 Oral history interview with Jane Hutcheson by author, 4 July 2019.

